# Increased Interleukin-11 Levels Are Correlated with Cardiac Events in Patients with Chronic Heart Failure

**DOI:** 10.1155/2019/1575410

**Published:** 2019-01-08

**Authors:** Jing Ye, Zhen Wang, Di Ye, Yuan Wang, Menglong Wang, Qingwei Ji, Ying Huang, Ling Liu, Ying Shi, Lei Shi, Tao Zeng, Yao Xu, Jianfang Liu, Huimin Jiang, Yingzhong Lin, Jun Wan

**Affiliations:** ^1^Department of Cardiology, Renmin Hospital of Wuhan University, Cardiovascular Research Institute, Wuhan University, Hubei Key Laboratory of Cardiology, Wuhan 430060, China; ^2^Department of Cardiology, The People's Hospital of Guangxi Zhuang Autonomous Region, Nanning, China

## Abstract

**Background:**

Interleukin-11 (IL-11) is an important inflammatory cytokine and has been demonstrated to participate in cardiovascular diseases. However, there have been no studies about the role of IL-11 in heart failure (HF). The present study is aimed at investigating whether IL-11 levels are associated with the cardiac prognosis in patients with HF.

**Methods:**

The plasma concentrations of IL-11 were measured in 240 patients with chronic HF (CHF) and 80 control subjects without signs of significant heart disease. In addition, we prospectively followed these CHF patients to endpoints of cardiac events.

**Results:**

Compared with the control group, the plasma IL-11 concentrations were significantly increased in the CHF patients and gradually increased in the New York Heart Association (NYHA) functional class II group, the NYHA functional class III group, and the NYHA functional class IV group. The receiver operating characteristic (ROC) curve revealed that the predictive role of IL-11 in HF is not as good as N-terminal B-type natriuretic peptide (BNP), although IL-11 has a certain value in predicting cardiac events. In addition, the CHF patients were divided into 3 groups according to the plasma IL-11 concentration category (low, T1; middle, T2; and high, T3). The multivariate Cox hazard analysis showed that the high plasma IL-11 concentrations were independently associated with the presence of cardiac events after adjustment for confounding factors. Furthermore, the CHF patients were divided into two groups based on the median plasma IL-11 concentrations. The Kaplan-Meier analysis revealed that the patients with high IL-11 concentrations had a higher risk of cardiac events compared with those with low IL-11 concentrations.

**Conclusions:**

Higher plasma IL-11 levels significantly increase the presence of cardiac events and suggest a poor outcome; although the diagnostic value of IL-11 in CHF is not as good as BNP, there is a certain value in predicting cardiac events in CHF.

## 1. Background

Heart failure (HF) is the final stage of all heart diseases, which lead to variety of serious clinical complications, including sudden cardiac death, the most dangerous complication. Although the clinical use of angiotensin-converting enzyme inhibitors/angiotensin receptor blocker (ACEI/ARB), *β*-blockers, and aldosterone receptor inhibitors is an effective method for improving survival rates, the prognosis of HF is still very poor and remains a major cause of death worldwide [1–3].

The previous studies had demonstrated that several interleukin-6 (IL-6) superfamily members were closely related to HF, such as leukemia inhibitory factor (LIF), oncostatin M (OSM), ciliary neurotrophic factor (CNTF), and cardiotrophin-1 (CT-1). A scientific statement from the American Heart Association indicated that IL-6 was an important biomarker of CHF [4]. An early literature reported that LIF mRNA levels were significantly increased in end-stage heart disease tissue [5]. In a later study, Gruson et al. reported that plasma OSM levels were significantly increased in CHF patients compared with healthy subjects but were not associated with the severity of left heart function [6]. In another study, Kreusser et al. demonstrated that CNTF participated in the progression of HF in a rat model [7]. In the recent study, Zhou et al. reported that microRNA-340-5p downregulated CT-1 expression and regulated the progression of HF [8].

IL-11 is a member of the IL-6 family along with IL-6, IL-31, LIF, OSM, CNTF, CT-1, cardiotrophin-2 (CT-2), and cardiotrophin-like cytokine (CLC) [9]. IL-11 is a multifunctional cytokine and takes part in various diseases by upregulating or downregulating inflammatory responses according to different inflammatory microenvironments [10, 11]. IL-11 has become a hot topic in the recent years as it had been reported to be involved in the onset and progression of multiple cancers and even directly affecting survival rates in cancer patients [12, 13]. The previous evidence suggested that IL-11 plays an important role in cardiovascular diseases. It was reported that cardiac myocytes are the target of IL-11, which could in turn alleviate hydrogen peroxide-induced cell death by activating the STAT3 pathway [14]. Later studies have demonstrated that the STAT3 pathway mediates the protective effects of IL-11 on ischemic cardiomyopathy in a mouse model, including acute myocardial infarction (AMI) and ischemia reperfusion injury (IRI) [15, 16]. IL-11 levels were observed to increase in human coronary artery disease (CAD) and aortic dissection (AD) [17, 18]. However, the circulating IL-11 levels in HF are still unknown. This study is aimed at clarifying the impact of plasma IL-11 concentrations on cardiac prognosis in HF patients.

## 2. Materials and Methods

### 2.1. Study Population

Consecutive patients (*N* = 240) who were admitted to People's Hospital of Guangxi Zhuang Autonomous Region between December 2013 and July 2014 for the treatment of worsening CHF, diagnosis and pathophysiological investigations, or for therapeutic evaluation of CHF were enrolled in this study. CHF was diagnosed by a cardiologist who had more than 25 years of clinical experience according to the guideline. A diagnosis of HF was based on a history of dyspnea and symptoms of exercise intolerance followed by pulmonary congestion, pleural effusion, or left ventricular enlargement by chest X-ray, or echocardiography was also considered in the diagnosis of CHF and in determining the left ventricular ejection fraction (LVEF) value [19, 20]. Individuals without significant heart disease, including significant CAD, systolic and diastolic dysfunction, valvular heart disease, or myocardial hypertrophy on echocardiography, were enrolled as control subjects (*N* = 80). All patients or their families provided informed consent. The study protocols were approved by the Medical Ethical Committee of the People's Hospital of Guangxi Zhuang Autonomous Region. The procedures were performed in accordance with the Helsinki Declaration.

### 2.2. Measurement of Plasma IL-11 Concentrations

Fasting venous peripheral blood samples were collected in Vacutainer tubes containing sodium heparin by nurses who had worked for more than 10 years. The samples were centrifuged for 20 min at 3000*g*, after which the supernatant was collected and stored at −80°C. Until the beginning of the experiments, the plasma samples were thawed at room temperature and the plasma IL-11 concentrations of all samples were measured using the enzyme-linked immunosorbent assay (ELISA) kits following the manufacturer's instructions (EHC128, NeoBioscience).

### 2.3. Measurement of Left Ventricular Structure and Function

The structure and function of the left ventricular of each patient were measured as described in the previous study [21].

### 2.4. Endpoints and Follow-Up

After discharge, the patients were asked to come to our hospital for a reexamination once a month by telephone and the follow-up was conducted by a cardiologist who had more than 10 years of clinical experience. The incidence of cardiac events was recorded. The follow-up ended according to incidence of either one of the cardiac events, including death due to progressive HF, myocardial infarction, stroke and sudden cardiac death, and rehospitalization for worsening HF. Sudden cardiac death was defined as death without definite premonitory symptoms or signs and was confirmed by two attending physicians. Doctors who participated in the follow-up had no prior knowledge of the measurement results of IL-11. The follow-up period for these patients was 13 (6 to 36) months.

### 2.5. Statistical Analyses

Data on the clinical characteristics and plasma IL-11 concentrations were expressed as the median (lower quartile to upper quartile) and were compared by the nonparametric test. The categorical variables are presented as counts (percentages). Spearman's correlation analysis was used to calculate correlations between BNP, LVEF, and plasma IL-11 concentrations. The receiver operating characteristic (ROC) curve was applied for predicting the diagnostic value of HF and cardiac event. Univariate and multivariate analyses with Cox proportional hazard regression were used to determine whether IL-11 was a significant predictor of cardiovascular events. The Kaplan-Meier method and log-rank test were used to compute and analyze the cumulative overall and event-free survival rates, respectively. A *P* value <0.05 was considered significant and was the threshold used to reject the null hypothesis.

## 3. Results

### 3.1. Comparison between Patients with and without CHF

The patient's blood samples were collected and sent to the clinical laboratory to test the expression of NT-pro BNP and other biochemical indicators. The results of all the biochemical indicators were collected from the reports of the clinical laboratory. Higher fasting glucose (Glu), creatinine (CREA), C-reactive protein (CRP), NT-pro BNP, and left ventricular end-diastolic dimension (LVEDD) and lower body mass index (BMI), blood pressure, albumin, and left ventricular ejection fraction (LVEF) were observed in CHF patients compared to the control subjects. Importantly, no significant differences for other characteristics, including age, gender, smoking, heart rate (HR), total cholesterol (TC), total triglycerides (TG), high-density lipoprotein cholesterol (HDL-C), and low-density lipoprotein cholesterol (LDL-C) were found between the two groups. In addition, the etiology of CHF in different NYHA functional classes displayed no difference. The clinical data of all the patients are listed in Table 1.

### 3.2. Plasma IL-11 Concentrations and CHF Severity

**Figure 1:**
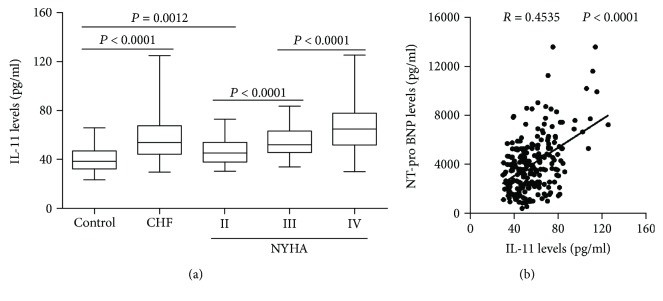
Plasma IL-11 concentrations in control subjects and CHF patients. (a) IL-11 levels in the control, CHF, NYHA II, NYHA III, and NYHA IV groups. *N* = 80 for the control group, *N* = 240 for the CHF group, *N* = 70 for the NYHA II group, *N* = 76 for the NYHA III group, and *N* = 94 for the NYHA IV group. (b) Correlation between the NT-pro BNP and IL-11 levels.

When compared with the control subjects, the plasma IL-11 concentrations were higher in the CHF group (Figure 1(a)). According to the New York Heart Association (NYHA) classification, the CHF patients were divided into NYHA II, NYHA III, and NYHA IV groups and the results showed that the plasma IL-11 concentrations were gradually increased in the NYHA II, NYHA III, and NYHA IV groups (Figure 1(a)). In addition, a correlation analysis showed that the plasma IL-11 concentrations were positively correlated with the NT-pro BNP levels (Figure 1(b)).

### 3.3. Comparison of IL-11 Concentrations with and without Cardiac Events

**Figure 2:**
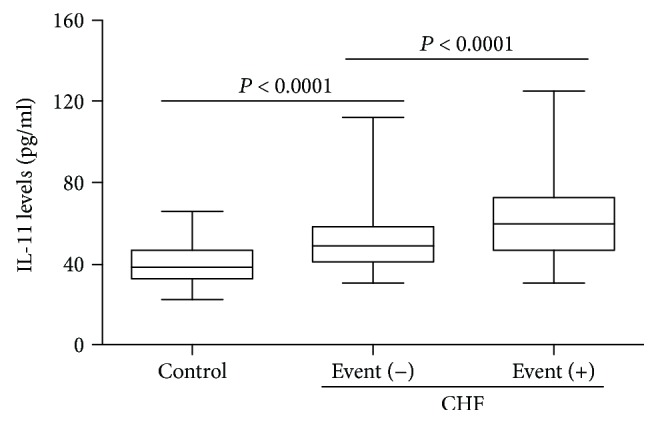
Comparisons of plasma IL-11 concentrations between the control subjects and HF patients with or without cardiac events. *N* = 80 for the control group, *N* = 102 for the CHF with cardiac event-free group, and *N* = 138 for the CHF with cardiac event group.

According to the results of the follow-up, there were 138 cardiac events including 22 deaths and 116 rehospitalizations in these CHF patients. We divided these CHF patients into a cardiac event-free group (event (−)) and a cardiac event group (event (+)). As shown in Table 2, with the exception of gender, no significant differences for other clinical characteristics were observed between these two groups. Moreover, patients with cardiac events showed markedly higher plasma IL-11 concentrations compared to those without cardiac events (Figure 2).

### 3.4. Association between Plasma IL-11 Concentrations and Cardiac Events

**Figure 3:**
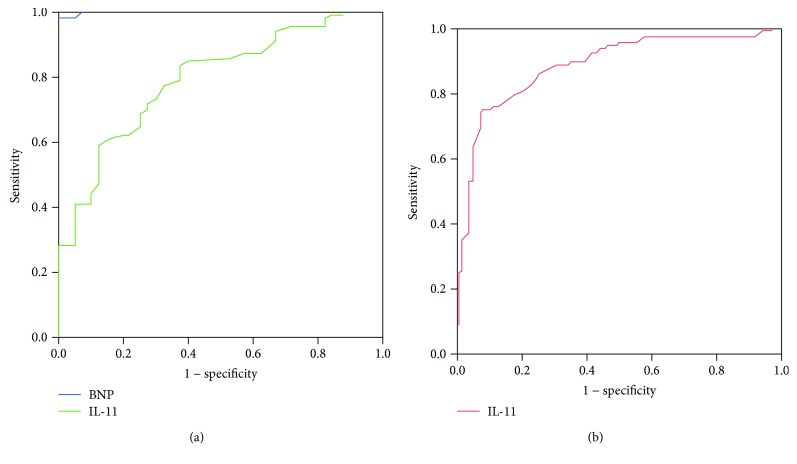
The diagnostic value of IL-11 in HF and cardiac event. (a) ROC curve of NT-pro BNP and IL-11 for predicting the diagnostic value of HF. (b) ROC curve of IL-11 for predicting the diagnostic value of cardiac event in CHF patients.

**Figure 4:**
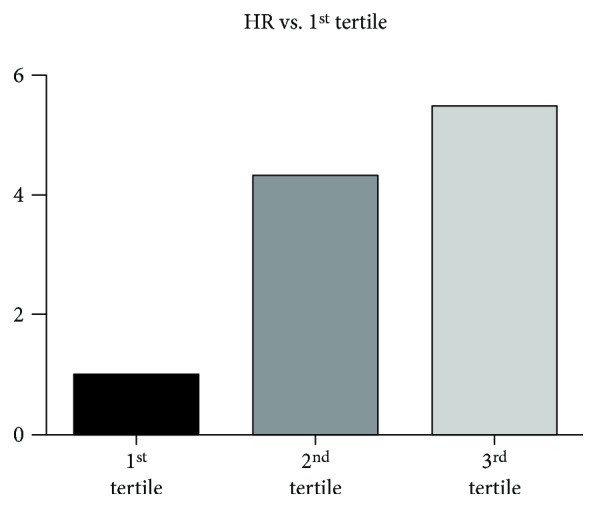
Hazard ratio of the tertiles of plasma IL-11 concentrations for cardiac events. *N* = 80 for each group.

**Figure 5:**
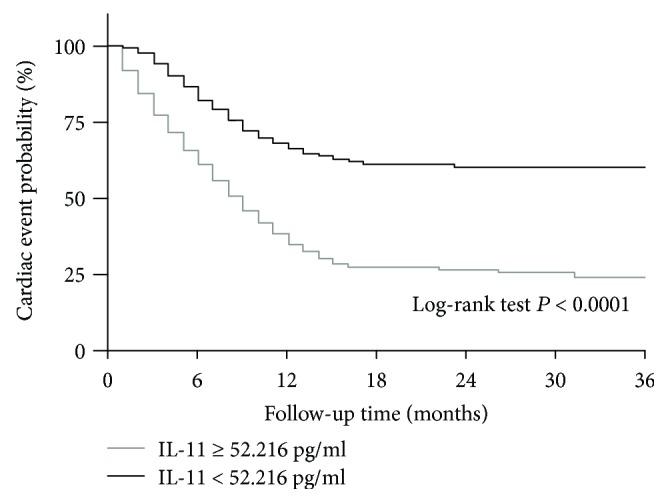
Kaplan-Meier curve for adverse cardiac events based on low and high plasma IL-11 concentrations. *N* = 120 for each group. *P* values are from the log-rank test.

The ROC curve revealed that the area under the curve (AUC) of BNP for predicting the positive outcome of HF was 0.999 (95% CI: 0.998-1.000, *P* < 0.0001; Figure 3(a)), while the AUC of IL-11 for predicting the positive outcome of HF was 0.825 (95% CI: 0.771-0.878, *P* < 0.0001; Figure 3(a)); the diagnostic value of BNP in HF was better than IL-11 (*P* < 0.0001; Figure 3(a)). The AUC of IL-11 for predicting the positive outcome of cardiac event was 0.886 (95% CI: 0.845-0.927, *P* < 0.0001; Figure 3(b)); the optimal serum IL-11 cutoff point for predicting the positive outcome of cardiac event was 57.7 pg/ml with a sensitivity of 75.2% and a specificity of 92.0%. The CHF patients were divided into three groups according to their plasma IL-11 concentration category (low T1, middle T2, and high T3). Multivariate Cox hazard analysis showed that the high (T3) plasma IL-11 concentrations were independently associated with cardiac events after adjustment for age, gender, smoking, BMI, albumin, CREA, CRP, BNP, LVEF, and LVEDD (hazard ratio 5.493, 95% confidence interval 2.096-14.395, *P* < 0.001; Figure 4 and Table 3). In addition, we divided these CHF patients into two groups according to the median plasma IL-11 concentrations. Kaplan-Meier analysis revealed that the patients with high plasma IL-11 concentrations had a higher risk of cardiac events compared to those with low plasma IL-11 concentrations (log-rank test *P* < 0.0001, Figure 5).

## 4. Discussion

In the present study, we detected the plasma IL-11 concentrations in CHF patients. We found that compared with the control subjects, circulating IL-11 concentrations were significantly increased in the CHF patients and correlated with the severity of HF. In addition, IL-11 could be used for predicting cardiac events in CHF patients and the follow-up data showed that increased plasma IL-11 concentrations increased cardiovascular events in CHF patients.

There are 40 members of the interleukin (IL) family, several members were observed to participate in HF. Panahi et al. reported that treatment with IL-1*β* blocker slowed the progression of HF after acute myocardial infarction [22]. In another study, Th1/Th2 imbalance was an important cause of HF and increased IL-12 and IL-18 and decreased IL-4 levels were observed [23]. In addition, higher plasma IL-6, IL-9, and IL-22 and decreased IL-5 and IL-7 levels were found in HF patients [24–27], and increased IL-22 levels were even critical with the occurrence of cardiac event [27]. In the present study, we found that another IL member, IL-11, was significantly increased in CHF patients and increased IL-11 levels were associated with circulating BNP levels. These data indicated that IL-11 was closely related to CHF and further improved our understanding of the relationship between the IL family and HF. However, the molecular mechanisms by which IL-11 participates in HF remain unknown, and further studies are needed.

The occurrence and development of HF are a complicated pathological process and may be associated with a combination of inflammatory responses, oxidative stresses, and myocardial apoptosis. Although the molecular and cellular mechanisms of HF remain unknown, it is certain that cardiac inflammation and fibrosis are major pathophysiological mechanisms operating in HF, as serial biomarker measurements and imaging modalities such as cardiac magnetic resonance imaging (CMR) have been shown to be linked to progressive cardiac fibrosis in the recent studies [28–30]. The dynamic interplay between inflammation and fibrosis leads to the gradual deterioration of cardiac function and ultimately HF. This explains why HF is the final stage of all the heart diseases, regardless of etiology [31]. IL-11 is an inflammatory cytokine and could regulate the inflammatory response in the heart. The previous studies have demonstrated that IL-11 participates in AMI and IRI by regulating the inflammatory cytokine section [15, 16]. In CAD patients, IL-11 is even associated with the Gensini score, which can be used to estimate the severity of coronary stenosis [17]. In our recent study, IL-11 was observed to be mainly secreted by macrophages and was increased in plasma and aortas and increased IL-11 may participate in the onset of AD via regulating interferon-*γ* (IFN-*γ*) and IL-17 secretion [18]. In the present study, we found that the levels of proinflammatory factor IL-11 were positively correlated with the severity of HF. Importantly, high plasma IL-11 concentrations carried a higher risk of cardiac events. Although IL-11 does not benefit the gold standard BNP in regard to diagnosing HF, it is still useful for predicting cardiac events in CHF patients. Furthermore, IL-11 may represent a new target for the prevention of clinical cardiovascular events in HF patients.

The primary fibrins in the heart are collagen I and collagen III, which together account for >90% of the total fibrin, and are the predominant contributors to interstitial fibrosis in the progression of HF [32]. In the recent letter published in *Nature*, Schafer et al. reported that IL-11 upregulation was the dominant transcriptional response to TGF-*β*1 exposure and was required for its profibrotic effects; the knockout of the IL-11 receptor could reduce cardiac fibrosis, making IL-11 a new therapeutic strategy to treat fibrotic diseases [33]. According to the results of the follow-up, higher IL-11 levels are associated with more cardiac events in CHF patients. Therefore, we suggested that higher IL-11 levels in the heart may lead to more severe fibrosis, further increasing cardiac events in CHF patients.

Data from clinical experiments and animal studies had demonstrated that IL-11 could regulate the differentiation of both T helper cells 1 (Th1) and Th17, which were the subsets of CD4^+^ Th cells [32, 33]. In a report, Lin et al. found that the exogenous recombinant human IL-11 treatment reduced a rate of 67.7% in Th1 and T-bet levels and normalized Th1/Th2 ratios in patients with immune thrombocytopenia [34]. Another study reported that in multiple sclerosis patients, IL-11 could induce a differentiation of naive CD4^+^ Th cells into Th17 cells, as well as the expansion of Th17 memory cells [35]. In the recent study, Cai et al. demonstrated that from the Th1/Th2 imbalance towards Th1 and from the Th17/Treg imbalance towards Th17, the cells were closely related to CHF and associated with the types, severity, and prognosis of the diseases [36]. In addition, according to the description above, the IL-6 family members, including OSM and SNTF, were demonstrated to significantly increase in plasma from HF patients [5–8]. These evidences suggested that inflammatory factors were closely related to HF. In the present study, we found that plasma IL-11 levels were also increased in CHF patients; our study further suggests that the IL-6 family members are strongly associated with the presence of HF.

In summary, the results of our study demonstrated that plasma IL-11 concentrations were dramatically increased in CHF patients and that increased plasma IL-11 levels were associated with the severity of left ventricular dysfunction. Although the exact mechanism is unknown, regulating inflammation is a possible mechanism. The present study had certain limitations. First, it was hard for us to collect the blood samples of HF with NYHA I, due to a lack of symptoms and a low diagnostic rate, although the cardiac structure had been changed and the heart function impaired. Therefore, we failed to enroll HF patients with NYHA I in the present study, and the plasma IL-11 levels were unclear. In addition, the role of IL-11 in HF and the mechanisms by which it operates remain uncertain, and further studies are needed to investigate these topics.

## 5. Conclusions

IL-11 is associated with the occurrence of cardiac events in CHF patients and may be a target for clinical prediction and prevention of cardiac events.

## Figures and Tables

**Table 1:** Information of clinical characteristics in the control and CHF group.

Characteristics	Control	CHF			
		Total	NYHA II	NAHY III	NYHA IV
Age (years)	53 (42, 58)	63 (56, 72)^∗^	63 (58, 72)^∗^	63 (57, 68)^∗^	61 (52, 73)^∗^
Male (*n*, %)	44 (55.0)	132 (55.0)	42 (60.0)	42 (55.3)	48 (51.1)
Smoking (*n*, %)	24 (30.0)	63 (26.3)	16 (22.9)	20 (26.3)	27 (28.7)
BMI (kg/m^2^)	24.7 (22.3, 27.3)	23.4 (21.6, 24.9)^∗^	24.1 (22.3, 25.3)	23.6 (22.4, 25.2)	22.4 (20.6, 24.5)^∗^^,#,†,§^
HR (bpm)	72 (68, 75)	69 (63, 75)	69 (64, 75)	71 (63, 77)	68 (63, 73)^∗^
SBP (mmHg)	125 (116, 133)	115 (105, 125)^∗^	113 (106, 123)^∗^	115 (105, 124)^∗^	117 (105, 128)^∗^
DBP (mmHg)	78 (72, 84)	74 (66, 79)^∗^	71 (64, 78)^∗^	73 (67, 76)^∗^	75 (67, 82)^∗^^,†^
TC (mmol/l)	4.2 (3.7, 4.7)	4.1 (3.5, 4.6)	4.1 (3.6, 4.6)	4.2 (3.6, 4.7)	4.1 (3.2, 4.5)^∗^^,§^
TG (mmol/l)	1.1 (0.8, 1.4)	1.0 (0.8, 1.3)	0.9 (0.7, 1.1)^∗^	1.0 (0.8, 1.4)	1.0 (0.8, 1.5)^†^
HDL-C (mmol/l)	1.0 (0.8, 1.3)	0.9 (0.8, 1.2)	1.0 (0.8, 1.2)	1.0 (0.8, 1.2)	0.9 (0.7, 1.1)
LDL-C (mmol/l)	2.0 (1.3, 2.5)	1.9 (1.4, 2.6)	1.9 (1.4, 2.3)	2.0 (1.4, 2.6)	1.9 (1.5, 2.6)
Glu (mmol/l)	5.3 (4.8, 6.0)	5.7 (4.9, 6.4)^∗^	5.2 (4.7, 6.1)^#^	5.9 (4.9, 6.5)^∗^^,†^	5.8 (5.0, 7.0)^∗^^,†^
Albumin (g/l)	43 (39, 47)	39 (35, 41)^∗^	39 (35, 41)^∗^	39 (36, 41)^∗^	38 (35, 41)^∗^
CREA (*μ*mol/l)	73 (64, 79)	92 (73, 124)^∗^	84 (68, 109)^∗^^,#^	93 (73, 126)^∗^^,†^	102 (82, 149)^∗^^,†^
CRP (mg/l)	0.5 (0.3, 1.0)	7.4 (3.2, 15.3)^∗^	5.8 (2.7, 9.7)^∗^^,#^	7.7 (3.3, 17.2)^∗^	11.3 (4.0, 18.3)^∗^^,†^
NT-pro BNP (pg/ml)	89 (74, 101)	3633 (2400, 5235)^∗^	3290 (1725, 4562)^∗^^,#^	3566 (2367,5220)^∗^	4138 (2528, 6236)^∗,#,†,§^
LVEF (%)	61 (56, 64)	39 (35, 42)^∗^	41 (37, 43)^∗^	38 (35, 42)^∗^^,†^	39 (35, 42)^∗^
LVEDD (mm)	47 (44, 50)	56 (52, 61)^∗^	53 (49, 57)^∗^^,#^	56 (53, 61)^∗^^,†^	56 (52, 63)^∗^^,†^
DCM (*n*, %)	-	68 (28.3%)	22 (31.4%)	20 (26.3%)	26 (27.7%)
IHD (*n*, %)	-	78 (32.5%)	20 (28.6%)	28 (36.8%)	30 (31.9%)
HHD (*n*, %)	-	62 (25.8%)	18 (25.7%)	20 (26.3%)	24 (25.3%)
Others (*n*, %)	-	32 (13.3%)	10 (14.3%)	8 (10.5%)	14 (14.9%)
Medications, (*n*, %)					
ACEI/ARB	0 (0%)	162 (67.5%)	38 (54.3%)^#^	52 (68.4%)	72 (76.6%)^§^
*β*-Blockers	4 (5.0%)	92 (38.3%)	34 (48.6%)	38 (50.0%)	20 (21.3%)^#,§,†^
Diuretics	0 (0%)	166 (69.2%)	34 (48.6%)^#^	48 (63.2%)	84 (89.4%)^#,§,†^
Digitalis	0 (0%)	166 (69.2%)	34 (48.6%)^#^	48 (63.2%)	84 (89.4%)^#,§,†^
Spironolactone	0 (0%)	118 (49.2%)	32 (45.7%)	28 (36.8%)^#,§^	58 (61.7%)^#,§,†^
Aspirin	8 (10%)	98 (40.8%)	22 (31.4%)	19 (23.7%)^#^	58 (61.7%)^#,§,†^
Statin	28 (35%)	72 (30.0%)	28 (40.0%)	20 (26.3%)	44 (46.8%)^#,†^

BMI: body mass index; HR: heart rate; SBP: systolic blood pressure; DBP: diastolic blood pressure; TC: total cholesterol; TG: total triglycerides; HDL-C: high-density lipoprotein cholesterol; HDL-C: low-density lipoprotein cholesterol; Glu: fasting glucose; CREA: creatinine; CRP: C-reactive protein; NT-pro BNP: NT-pro brain natriuretic peptide; LVEF: left ventricular ejection fraction; LVEDD: left ventricular end-diastolic dimension; DCM: dilated cardiomyopathy; IHD: ischemic heart disease; HHD: hypertensive heart disease; ACEI: angiotensin-converting enzyme inhibitor; ARB: angiotensin receptor blocker. ^∗^*P* < 0.05 vs. the control group. ^#^*P* < 0.05 vs. the total CHF group. ^§^*P* < 0.05 vs. the NYHA II group. ^†^*P* < 0.05 vs. the NYHA III group.

**Table 2:** Comparison of patients with or without cardiac event in CHF patients.

Characteristics	Cardiac event (−)	Cardiac event (+)	*P* value
Age (years)	63 (55, 71)	63 (57, 72)	0.889
Male (*n*, %)	65 (63.7)	67 (48.6)	0.026
Smoking (*n*, %)	32 (31.4)	30 (21.8%)	0.184
BMI (kg/m^2^)	23.5 (21.7, 25.2)	23.3 (21.4, 24.8)	0.278
HR (bpm)	71 (63, 75)	68 (63, 76)	0.375
SBP (mmHg)	114 (104, 125)	115 (107, 126)	0.251
DBP (mmHg)	73 (65, 78)	75 (67, 80)	0.160
TC (mmol/l)	4.1 (3.6, 4.6)	4.1 (3.5, 4.6)	0.599
TG (mmol/l)	1.0 (0.8, 1.3)	1.0 (0.5, 1.3)	0.914
HDL-C (mmol/l)	1.0 (0.7, 1.3)	0.9 (0.8, 1.1)	0.898
LDL-C (mmol/l)	2.0 (1.5, 2.5)	1.9 (1.4, 2.6)	0.582
Glu (mmol/l)	5.7 (4.9, 6.6)	5.4 (4.9, 6.4)	0.252
Albumin (g/l)	39 (35, 42)	38 (35, 41)	0.285
CREA (*μ*mol/l)	92 (73, 120)	92 (73, 124)	0.965
CRP (mg/l)	9.6 (4.7, 18.4)	6.0 (2.6, 14.8)	0.005
NT-pro BNP (pg/ml)	3763 (2452, 5224)	3573 (2349, 5253)	0.897
LVEF (%)	39 (35, 42)	39 (35, 42)	0.299
LVEDD (mm)	54 (50, 59)	56 (52, 62)	0.047
NYHA functional class, II/III/IV	28/34/40	42/42/54	0.783/0.791/0.994
DCM (*n*, %)	32 (31.4%)	36 (26.1%)	0.545
IHD (*n*, %)	36 (35.3%)	38 (27.5%)	0.426
HHD (*n*, %)	22 (21.6%)	40 (29.0%)	0.404
Others (*n*, %)	20 (19.6%)	16 (11.6%)	0.302
Medications, (*n*, %)			
ACEI/ARB	76 (74.5%)	86 (62.3%)	0.052
*β*-Blockers	36 (35.3%)	56 (40.6%)	0.423
Diuretics	61 (59.8%)	86 (62.3%)	0.789
Digitalis	72 (70.6%)	94 (68.1%)	0.887
Spironolactone	46 (45.1%)	72 (52.2%)	0.241
Aspirin	44 (43.1%)	54 (39.1%)	0.598
Statin	29 (28.4%)	32 (23.2%)	0.454

**Table 3:** Association between IL-11 and the presence of cardiac events was assessed by univariate and multivariate analyses.

	Univariate analysis			Multivariate analysis		
	HR	95% CI	*P* value	HR	95% CI	*P* value
Low	1	Reference	Reference	1	Reference	Reference
Middle	4.761	2.076 to 13.114	<0.001	4.340	1.599 to 11.781	0.004
High	6.314	1.463 to 17.423	<0.001	5.493	2.096 to 14.395	0.001

## Data Availability

We declared that materials described in the manuscript, including all relevant raw data, will be freely available to any scientist wishing to use them for noncommercial purposes, without breaching participant confidentiality.
